# Acceptance towards decriminalization of medical marijuana among adults in Selangor, Malaysia

**DOI:** 10.1371/journal.pone.0262819

**Published:** 2022-02-10

**Authors:** Rahmat Dapari, Mohd Hafizuddin Mahfot, Ahmad Iqmer Nashriq Mohd Nazan, Mohd Rohaizat Hassan, Nazri Che Dom, Syed Sharizman Syed Abdul Rahim

**Affiliations:** 1 Department of Community Health, Faculty of Medicine and Health Sciences, Universiti Putra Malaysia, Serdang, Selangor, Malaysia; 2 Department of Community Health, Faculty of Medicine, National University of Malaysia, Cheras, Kuala Lumpur, Malaysia; 3 Faculty of Health Sciences, Universiti Teknologi MARA, Puncak Alam, Selangor, Malaysia; 4 Public Health Medicine Department, Faculty of Medicine and Health Sciences, Universiti Malaysia Sabah, Kota Kinabalu, Sabah, Malaysia; UCSI University, MALAYSIA

## Abstract

**Introduction:**

The issue of decriminalization of medical marijuana has gained public attention globally due to the decisions of various governments in developed and developing countries who have decriminalized marijuana for medical purposes. The action was the result of the change in perception towards medical marijuana use, which is now believed to be safe, acceptable, and should be decriminalized. Due to the progress of modernization and the wide access to information, the change in perception towards medical marijuana seems to be inevitable and might have already permeated among the public in Malaysia. However, at the moment there is no baseline data to determine any of this claim.

**Objective:**

To determine the prevalence and factors associated with acceptance towards decriminalization of medical marijuana among adults in Selangor, Malaysia.

**Methodology:**

The study was a cross-sectional study conducted in year 2021 among 462 adults aged 18 years old and above in Selangor, Malaysia. The respondents were sampled using a multistage random sampling. The data was collected via self-administered questionnaires and has been analyzed using SPSS version 25.

**Result:**

More than half of the respondents in this study (64.7%) show acceptance towards the decriminalization of medical marijuana in Malaysia. The results of statistical tests indicate that there are significant associations between age (p < 0.001), gender (p = 0.005), ethnicity (p < 0.012), level of education (p < 0.011), employment status (p = 0.001), ever smoked (p < 0.001), given up smoking (p = 0.002), ever used substance (p < 0.001), current substance use (p < 0.001), given up substance (p < 0.001), exposure to medical marijuana-related content (p < 0.001), perceived risk associated with medical marijuana use (p < 0.001), perceived risk of harm of medical marijuana use (p < 0.001), and perceived approval of medical marijuana use (p < 0.001) with acceptance towards decriminalization of medical marijuana. The predictors for acceptance towards decriminalization of medical marijuana are perceived high approval of medical marijuana use (aOR = 7.023, p < 0.001, 95%CI = 3.534,13.955), perceived low risk of medical marijuana (aOR = 5.716, p < 0.001, 95%CI = 2.828,11.554), perceived low risk of harm from medical marijuana use (aOR = 3.480, p = 0.001, 95%CI = 1.702,7.114), current substance use (aOR = 2.264, p = 0.050, 95%CI = 1.001,5.118), and ever used substance (aOR = 2.005, p = 0.004, 95% CI = 0.054,0.576).

**Conclusion:**

The results of the survey show that the current acceptance level towards decriminalization of medical marijuana is considerably high. However, the acceptance is mainly among those who are exposed to the substance and those who perceive low risk of medical marijuana. Thus, a further exploration of this phenomenon is needed, especially by increasing the sample size and expanding the study location to other states.

## Introduction

Based on the written evidence from ancient botanical history and traditional texts on herbal medicine, marijuana has been recorded to possess specific medicinal properties and has been used historically to treat medical symptoms such as chronic pain and seizure [1]. However, the use of marijuana is much more commonly associated with recreational use, crime, and social problems [2]. Thus, in this study, the term medical marijuana is applied to refer to the use of marijuana specifically for medical purposes.

Marijuana was listed under Schedule IV of the 1961 Single Convention on Narcotic Drugs by the United Nations Commission on Narcotic Drugs (CND) alongside heroin, morphine, and cocaine before being delisted on December 2, 2020 [3]. In Malaysia, marijuana is listed in the First Schedule [Sections 2, 11 (1) and 17 (30)] under the Dangerous Drugs Act 1952 (Act 234) where ingestion and possession of marijuana in any form is illegal and a crime and possession of marijuana of more than 200 g despite the intention of use is a severe criminal act punishable by death. Malaysia had been committed to the fight against drugs and substance abuse long before the announcement of the Country’s Number One Enemy campaign in 1983 [4]. However, from 2018 to 2019, there was an increase of 8% in the number of drug and substance abusers and addicts in Malaysia [5, 6].

Decriminalization, which is defined as removing criminal sanctions against an act, article, or behaviour (Svrakic et al., 2012), has been advocated to be the better approach to tackling drug-related issues than the war on drugs. This notion stems from the growing perception that the long, arduous journey of criminalization through the declaration of the war on drugs has been a failure, leading to more violence and corruption [2].

Decriminalizing drugs would mean that though the prohibited drug will remain illegal, the person in question will not be severely prosecuted by law for possession or administration of drugs. Instead, the person would be punished with fines, community service or undergo drug treatment. In essence, a less severe punishment than previously legislated [7]. In Canada, specifically for medical marijuana, the decriminalization of the substance came in a form of expansion of medical services where patients may gain access to medical marijuana through medical dispensaries under strict measures [8].

The approach of decriminalization of drugs has been adopted by more than 25 countries worldwide with significant differences and levels of effectiveness. Among countries that have been adopting decriminalization approaches are Germany, Italy, Switzerland, the Czech Republic, and Portugal, with decriminalization models varying from one country to another to suit the local context [2].

The Malaysian government had previously considered decriminalization in a form of a dual-policy approach. However, the focus was more on hard drugs such as heroin and morphine, in which the primary usage method is through intravenous injection, a method posing high risks of transmitting HIV. In this case, people who inject drugs (PWID) were given free syringes through the National Syringe Exchange Programme (NSEP) or enrolment in the Methadone Replacement Therapy Programme (MRT) [9].

In the case of marijuana, the issue of decriminalization for medical purposes is related to providing legal access for patients to seek medication in the form of medical marijuana to treat their symptoms or illness, whenever supporting clinical and scientific evidence is available. The exposure to medical marijuana-related information that is widely spread through online media may have influenced how marijuana is being perceived currently, for its risk as a substance, risk of harm of use, and approval of use, resulting in it being reported less to NADA. In the US, these components have been monitored among adults since 1975 through a national survey on drug use alongside the other hard drugs. The survey showed that over time marijuana use is more commonly being perceived to be of low risk, low risk of harm, and to have high approval of use [10]. Together with other supporting data, marijuana has now been decriminalized in 34 states in the U.S. for medical purposes. However, repetitive exposure to certain concepts from online media sources can easily sway people into perceiving those concepts as true even though they might not be; this change in perception was conceptualized in the cultivation theory by George Gerbner in the 1960s [11].

The recent decision by the United Nations Commission on Narcotic Drugs to remove marijuana from Schedule IV of the 1961 Single Convention on Narcotic Drugs, in which it was previously listed alongside deadly, addictive opioids, including heroin is expected to spur discussion among the public on the current Malaysian government policy on medical marijuana use. Together with the increasing trend of decriminalization of medical marijuana globally, the public may perceive marijuana not to be as harmful as previously thought. However, there is no current data available on the perception of such components, and the prevalence of acceptance towards decriminalization of marijuana especially among the adult population in our local Malaysian setting.

In the case of medical marijuana, though there is still limited scientific evidence of its medicinal properties in treating various diseases, the claims of its benefits are widely shared, discussed, and promoted among the public. The information on how different countries, especially developed countries, have different policy approaches towards the use of the substance, has also opened a window of discussion among the public. Comparison is inevitable, making the public question the current criminalization approach of the government in handling medical marijuana issues. In Malaysia, as of 2017, there were 1,122 prisoners on death row with 71% of them convicted of drug trafficking which includes marijuana [12].

At the same time, there are no legal procedures provided for qualified patients to get access to medical marijuana in Malaysia. This contrasts with the situation in Canada, where access to medical marijuana is available for patients through medical dispensaries via strict measures and focuses on a limited number of medical conditions such as end-of-life care, multiple sclerosis, cancer, spinal cord injury, HIV/AIDS infection, a severe form of arthritis, and epilepsy [8]. In Germany, medical marijuana can be prescribed by a medical professional in situations where recognized standard treatment options are not available and the prospect of the impact of medical marijuana therapy is not entirely implausible [13]. The situation is similar in Thailand where the use of marijuana for medical reasons is allowed with a prescription from either medical doctor, dentist, or registered Thai-traditional medical personnel [14].

The aim of this study is to determine the prevalence and factors associated with acceptance towards decriminalization of medical marijuana among adults in Selangor, Malaysia. The results can be used to formulate balanced measures between strategic intervention and the possibility of medical services expansion in terms of access to medical marijuana treatment for affected patients similar to what can be learned from Canada, Germany, and Thailand [15].

## Methods

### Study design and location

This cross-sectional study was performed in Selangor, Malaysia. The inclusion criteria for this study were Malaysians aged 18 years and above, residing in Selangor, Malaysia. Selangor has the lowest number of substance abusers and drug addicts per estimated total population (210 per 100,000 residents) compared with other states, despite being the most populous state in Malaysia as shown in Table 1 [16].

**Table 1:** Number of drug and substance abusers and addicts according to state in Malaysia and estimated total population in 2019.

STATE	NUMBER OF DRUG AND SUBSTANCE ABUSERS AND ADDICTS	%	ESTIMATED TOTAL POPULATION (’000)	RATIO OF DRUG AND SUBSTANCE ABUSER AND ADDICTS PER ESTIMATED TOTAL POPULATION	NUMBER OF DRUG AND SUBSTANCE ABUSERS AND ADDICTS PER 100,000 POPULATION
Johor	16,124	11.34	3,764.30	0.43	428
Kedah	11,629	8.18	2,180.60	0.53	533
Kelantan	16,341	11.49	1,885.70	0.87	867
Melaka	4,968	3.49	930.7	0.53	534
Negeri Sembilan	7,934	5.58	1,130.30	0.7	702
Pahang	13,616	9.58	1,674.60	0.81	813
Pulau Pinang	8,987	6.32	1,774.60	0.51	506
Perak	9,335	6.56	2,512.10	0.37	372
Perlis	2,064	1.45	254.4	0.81	811
Selangor	13,722	9.65	6,528.40	0.21	210
Terengganu	11,409	8.02	1,245.70	0.92	916
Sabah*	10,886	7.66	4,002.70	0.27	272
Sarawak	6,774	4.76	2,812.80	0.24	241
WP Kuala Lumpur**	8,410	5.91	1,884.50	0.45	446
Total	142,199	100	32,581.40	0.44	436

### Sample-size calculation

We estimated the required sample size for each potentially associated factor and used the highest estimated number. The sample size for this study was calculated using the two proportions formula. The required sample size for this study with 95% precision and 80% power, was estimated to be 398 based on study attitudes toward medical cannabis legalization [17]. For the online survey, a 30% drop out rate was added and the final sample size required became 517.

### Study instruments and data collection

A self-administrated questionnaire in two language versions (English and Malay) in Google Form was used in this study. The questionnaire was divided into three sections. Section 1 consists of 21 mixed questions which collected data relevant to sociodemographic, socioeconomic, and lifestyle factors, and was adapted from a study on support for marijuana legalization and predictors of intentions to use marijuana more often in response to legalization among U.S. young adults [18]. Section 2 consists of 10 questions using a 5-point Likert scale to assess the perception of medical marijuana. The perceived risk of medical marijuana component was adapted from a study on characteristics associated with attitudes towards marijuana legalization in Michigan [19]. The perceived risk of harm of medical marijuana uses and perceived approval of use of medical marijuana component was adapted from the study on support for marijuana legalization and predictors of intentions to use marijuana more often in response to legalization among U.S. young adults [18]. Section 3 consists of five questions using a 5-point Likert scale to assess the overall acceptance towards decriminalization of medical marijuana in Malaysia, and was developed from the study on characteristics associated with attitudes toward marijuana legalization in Michigan [19].

The total population in Selangor is currently estimated at 6.53 million, with 3.40 million males and 3.13 million females. About 70% the population are adults of age 20 and above. There are 16 parliamentary and a total of 56 state legislative assembly constituents in Selangor. Each parliamentary constituent will have two to three state legislative assembly constituents. For data collection, a multistage probability random sampling technique was used to select six parliamentary and state legislative assembly constituents. Potential respondents were selected randomly from the selected constituent list using a randomization tool software, as per sample size calculation. The respondents were assessed for eligibility before being invited to participate in the study. This method was selected in order to obtain a workable sample size. Data collection was done gradually in June 2021.

### Face and content validity

For the face validity of this study, a pre-testing of the questionnaire was conducted involving 30 members of the community. For content validity, the questionnaire was evaluated by a subject matter expert to ensure that the items used in the instruments were aligned with the study objectives. The questionnaire was then translated to the Malay language to ensure reliability. The translated Malay language questionnaire was validated by a professional content writer and translator for its comprehensiveness and actual meaning in the Malay language. Modifications were done based on the advice of the supervisory committee and the expert on the matter.

### Reliability

The magnitude of the reliability of the study was assessed via a test-retest of the questionnaire that was conducted among 30 community members. For the reliability test, the average inter-item correlation (AIC) for the acceptance towards decriminalization of medical marijuana scale was 0.249 (-0.01 to 0.45) and Cronbach’s alpha was 0.708 after removing a total of 12 items from the scale. The desired AIC range to be 0.15 to 0.20 for measuring a broad higher-order construct. Similarly, most psychometricians agreed with the notion that a Cronbach’s alpha value of 0.70 is acceptable. Both the obtained AIC and alpha values proved the adequate internal consistency for the nine remaining items as shown in Table 2.

**Table 2:** Reliability test results of questionnaire items (N = 9).

Instrument	Mean	Std. Deviation	Cronbach’s Alpha
Ever drank	1.3667	.49013	.677
Given up drinking	1.9667	.88992	.763
Current substance use	1.9667	.18257	.705
Given up substance	2.9333	.25371	.711
Exposure to MMJ content	1.2333	.43018	.702
Perceived risk of MMJ	1.2667	.44978	.643
Perceived risk of harm	1.3000	.46609	.637
Perceived approval of use	1.3000	.46609	.653
Acceptance	1.2333	.43018	.655

### Data analysis

The collected data from the questionnaires were analysed using SPSS software (IBM SPSS Version 25.0). In descriptive statistics, the data is presented in mean and standard deviation if normally distributed and as median and interquartile range if not normally distributed. Descriptive statistics were used to describe the characteristics of the distribution of respondents by sociodemographics, socioeconomics, perception of medical marijuana, and acceptance towards decriminalization of medical marijuana.

All hypothesis testing in this study used a two-directional test with the significance level (α) set at 0.05. The Chi-square test was used to determine the association between the dependent variable and all the independent variables with categorical data. For variables with numerical data, the association was examined by using Independent T-test. All the significant findings in hypothesis testing were subsequently tested by using multiple logistic regression, to find the significant adjusted odds ratio at 95% confidence intervals.

### Ethical approval

Ethical approval was obtained from the Ethics Committee for Research involving Human Subject of Universiti Putra Malaysia (JKEUPM– 2021–203). Consent from each participant was obtained using an online consent form upon agreement to participate in the study before answering the questionnaire.

## Results

A total of 462 responses were received with an overall response rate of 89.4%. All the respondents were contacted via WhatsApp or Telegram application with the contact numbers obtained from the community representatives at the study location and confirmed with the name list of voters from the Malaysian Election Commission.

The results from the descriptive statistics are presented in Table 3. The age range of participants involved in this study was 19–67 year-old with an average age of 36.3 years (SD = 10.85). From the sample, the majority of the respondents were online media users (98.7%), Malay (82.5%), and male (76.2%).

**Table 3:** Descriptive statistics of the respondents based on sociodemographic, socioeconomic, and lifestyle factors, and perception with acceptance towards decriminalization of medical marijuana.

Variable	M	SD
Age (year)	36.3	10.85
Daily online media engagement (hours)	4.97	3.6
	**N**	**%**
Gender		
Male	352	76.2
Female	110	23.8
Ethnicity		
Malay	381	82.5
Chinese	39	8.4
Indian	20	4.3
Others	22	4.8
Level of education		
Primary school	3	0.6
Secondary school	100	21.6
STPM/Matriculation/Diploma	146	31.6
First degree (Bachelor)	168	36.4
Second/Third degree (Master/PhD)	45	9.7
Employment status		
Government servant	69	14.9
Private company	190	41.1
Self-employed	136	29.4
Student	21	4.5
Unemployed	46	10
Household Monthly income		
B40	205	44.4
M40	165	35.7
T20	92	19.9
Smoking		
Ever smoked		
Yes	317	68.6
No	145	31.4
Current cigarette smoke		
Yes	207	44.8
No	255	55.2
Given up smoking		
Yes	117	25.3
No	200	43.3
Never smoke	145	31.4
Alcohol		
Ever drank		
Yes	178	38.5
No	284	61.5
Current alcohol drinker		
Yes	40	8.7
No	422	91.3
Given up alcohol		
Yes	112	24.2
No	66	14.3
Never drank	284	61.5
Substance Use		
Ever used		
Yes	222	48.1
No	240	51.9
Current substance use		
Yes	122	26.4
No	340	73.6
Given up substance		
Yes	75	16.2
No	147	31.8
Never used drugs	240	51.9
Online media		
Online media user		
Yes	456	98.7
No	6	1.3
Exposure to marijuana-related content		
Yes	340	73.6
No	122	26.4
Perceived risk associated with medical marijuana		
Low risk	285	61.7
High risk	177	38.3
Perceived risk of harm of medical marijuana use		
Low risk	292	63.2
High risk	170	36.8
Perceived approval of use of medical marijuana average score		
High approval	269	58.2
Low approval	193	41.8
Acceptance towards decriminalization of medical marijuana		
Accept	299	64.7
Reject	163	35.3

Note: N = Number M = Mean, SD = Standard deviation.

Most of the respondents in this study have ever smoked (68.6%), perceived low risk of harm from medical marijuana use (63.2%), perceived low risk from medical marijuana (61.7%), never drank alcohol (61.5%), perceived high approval for medical marijuana use (58.2%), never used substance (51.9%), in B40 category for household monthly income (44.4%), employed by private companies (41.1%), and have a first degree (36.4%). The acceptance rate towards decriminalization of medical marijuana from the respondents in this study was observed to be 64.7%.

The results on the associations between acceptance towards decriminalization of medical marijuana and sociodemographic, socioeconomic, and lifestyle factors, and perception of medical marijuana use are presented in Table 4.

**Table 4:** Associations between acceptance towards decriminalization of medical marijuana and sociodemographic, socioeconomic, and lifestyle factors, and perception of medical marijuana use.

**Variable**	**N**	**Accept** M (SD)	**Reject** M (SD)	**t statistic (df)**		**p-value**	**95% CI**
**Age**	462	35 (10.6)	38.67 (10.8)	-3.521		**<0.001**	-5.726, -1.624
**Daily online media engagement (hours)**	462	5.4 (3.8)	4.18 (2.99)	3.797		**<0.001**	0.590, 1.857
**Variable**	**N (%)**	**Accept n (%)**	**Reject n (%)**	**X** ^**2**^	**df**	**p-value**	**95% CI**
**Gender**				7.766	1	**0.005**	
Male	352 (76.2)	240 (68.2)	112 (31.8)				1.269, 1.367
Female	110 (23.8)	59 (53.6)	51 (46.4)				1.369, 1.559
**Ethnicity**				11.015	3	**0.012**	
Malay	381 (82.5)	257 (67.5)	124 (32.5)				1.278, 1.372
Chinese	39 (8.4)	21 (53.8)	18 (46.2)				1.298, 1.625
Indian	20 (4.3)	7 (35.0)	13 (65.0)				1.421, 1.879
Others	22 (4.8)	14 (63.6)	8 (36.4)				1.145, 1.581
**Level of education**				13.113	4	**0.011**	
Primary school	3 (0.6)	1 (33.3)	2 (66.7)				0.232, 3.100
Secondary school	100 (21.6)	51 (51)	49 (49)				1.390, 1.590
STPM/Matriculation/Diploma	146 (31.6)	97 (66.4)	49 (51.5)				1.258, 1.413
First degree (Bachelor)	168 (36.4)	120 (71.4)	48 (28.6)				1.217, 1.355
Second/Third degree (Master/PhD)	45 (9.7)	30 (66.7)	15 (33.3)				1.190, 1.477
**Employment status**				18.638	4	**0.001**	
Government servant	69 (14.9)	44 (63.8)	25 (36.2)				1.246, 1.479
Private company	190 (41.1)	136 (71.6)	54 (28.4)				1.219, 1.349
Self-employed	136 (29.4)	85 (62.5)	51 (37.5)				1.292, 1.457
Student	21 (4.5)	16 (76.2)	5 (23.8)				1.039, 1.437
Unemployed	46 (10)	18 (39.1)	28 (60.9)				1.462, 1.755
**Household monthly income**				.434	2	0.805	
Less than RM4849	205 (44.4)	136 (66.3)	69 (33.7)				1.271, 1.402
RM4850-RM10959	165 (35.7)	105 (63.6)	60 (36.4)				1.289, 1.438
More than RM10960	92 (19.9)	58 (59.5)	34 (37.5)				1.269, 1.470
**Smoking**							
Ever smoked				12.486	1	**<0.001**	
Yes	317 (68.6)	222 (70.0)	95 (30.0)				1.249, 1.350
No	145 (31,4)	77 (53.1)	68 (46.9)				1.387, 1.551
Current cigarette smoker				3.127	1	0.077	
Yes	207 (44.8)	143 (69.1)	64 (30.9)				1.246, 1.373
No	255 (55.2)	156 (61.2)	99 (38.8)				1.328, 1.449
Given up smoking				12.709	2	**0.002**	
Yes	117 (25.3)	80 (68.4)	37 (31.6)				1.231, 1.402
No	200 (43.3)	142 (71.0)	58 (29.0)				1.227, 1.353
Never smoke	145 (31.4)	77 (53.1)	68 (46.9)				1.387, 1.551
**Alcohol**							
Ever drank				3.845	1	0.05	
Yes	178 (38.5)	125 (70.2)	53 (29.8)				1.229, 1.367
No	284 (61.5)	174 (61.3)	110 (38.7)				1.330, 1.444
Current alcohol drinker				1.161	1	0.281	
Yes	40 (8.7)	29 (72.5)	11 (27.5)				1.130, 1.419
No	422 (91.3)	270 (64.0)	152 (36.0)				1.314, 1.406
Given up alcohol				3.857	2	0.145	
Yes	112 (24.2)	79 (70.5)	33 (29.5)				1.208, 1.380
No	66 (14.3)	46 (69.7)	20 (30.3)				1.189, 1.417
Never drank	284 (61.5)	174 (61.3)	110 (38.7)				1.330, 1.444
**Substance Use**							
Ever used				10.120	1	**0.001**	
Yes	222 (48.1)	160 (72.1)	62 (27.9)				1.219, 1.338
No	240 (51.9)	139 (57.9)	101 (42.1)				1358, 1.484
Current substance use				47.007	1	**<0.001**	
Yes	122 (26.4)	110 (90.2)	12 (9.8)				1.045, 1.152
No	340 (73.6)	189 (55.6)	151 (44.4)				1.391, 1.497
Given up substance				64.101	2	**<0.001**	
Yes	75 (16.2)	57 (76.0)	18 (24.0)				1.141, 1.339
No	147 (31.8)	127 (86.4)	20 (13.6)				1.080, 1.192
Never used substance	240 (51.9)	115 (47.9)	125 (52.1)				1.457, 1.585
**Online media**							
Online media user				0.100	1	0.920	
Yes	456 (98.7)	295 (64.7)	161 (35.3)				1.309, 1.397
No	6 (1.3)	4 (664.7)	2 (33.3)				0.791, 1.875
**Exposure to marijuana-related content**				46.745	1	**<0.001**	
Yes	340 (73.6)	251 (73,8)	89 (26.2)				1.215, 1.309
No	122 (26.4)	48 (39.3)	74 (60.7)				1.518, 1,695
**Perceived risk of medical marijuana**				211.129	1	**<0.001**	
Low risk	285 (61.7)	257 (90.2)	28 (9.8)				1.064, 1.133
High risk	177 (38.3)	42 (23.7)	135 (76.3)				1.699, 1.826
**Perceived risk of harm of medical marijuana use**				199.848	1	**<0.001**	
Low risk	292	259 (88.7)	33 (11.3)				1.077, 1.150
High risk	170	40 (23.5)	130 (76.5)				1.700, 1.829
**Perceived approval of use of medical marijuana**				218.676	1	**<0.001**	
High approval	269 (58.2)	249 (92.6)	20 (7.4)				1.043, 1.110
Low approval	193 (41.8)	50 (25.9)	143 (74.1)				1.679, 1.803

Note: p-value < 0.05 is significant.

### Predictors of acceptance towards decriminalization of medical marijuana

Multiple logistic regression was performed to assess the impact of independent variables on the likelihood that respondents would report that they had an acceptance towards decriminalization of medical marijuana. The initial model contained 20 independent variables (age, gender, ethnicity, level of education, employment status, household monthly income, ever smoked, current smoking status, given up smoking, ever drank, current alcohol drinker, given up alcohol, ever used substance, current substance use, online media user, exposure to medical marijuana-related content, hours spent on online media, perceived risk of medical marijuana, perceived risk of harm of medical marijuana use, and perceived approval of medical marijuana use).

As shown in Table 5, only five of the independent variables made a statistically significant contribution to the model (ever used substance, current substance use, perceived low risk of medical marijuana, perceived low risk of harm of medical marijuana use, and perceived high approval of medical marijuana use) using “Forward LR” method.

**Table 5:** Multiple logistic regression analysis predicting acceptance towards decriminalization of medical marijuana in Malaysia.

Variable	B	SE	Wald	p-value	aOR	95% CI
Ever used substance	0.696	0.317	4.819	0.028	2.005	1.077–3.732
Current substance use	0.817	0.416	3.854	0.050	2.264	1.001–5.118
Perceived risk of medical marijuana	1.743	0.359	23.568	0.000	5.716	1.702–7.114
Perceived risk of harm of medical marijuana	1.247	0.365	11.682	0.001	3.480	3.534–13.955
Perceived approval of use	1.949	0.350	30.952	0.000	7.023	1.077–3.732
Constant	-2.444	0.309	62.672	0.000	.087	

Note: p-value < 0.05 is significant.

Hosmer and Lemeshow test, X^2^ (6) = 6.795, p = 0.340, Cox and Snell R^2^ = 0.494.

Nagelkerke R^2^ = 0.679.

The full model had a good fit, indicating that the model was able to distinguish between respondents who reported and did not report an acceptance towards decriminalization of medical marijuana. The model as a whole explained between 49.4% (Cox and Snell R^2^) and 67.9% (Nagelkerke R^2^) of the variance in acceptance towards decriminalization of medical marijuana, and correctly classified 91.3% of cases. Other variables were not significant predictors for acceptance towards decriminalization of medical marijuana.

## Discussion

Selangor has the lowest number of substance abusers and drug addicts per estimated total population compared with other states and this finding could be explained as those who reside in urban areas have a higher level of awareness on issues regarding drug abuse and addiction and its commensurate risks and danger [20].

The analysis of the data shows that age is significantly associated with acceptance towards decriminalization of medical marijuana (p < 0.001). Similar findings were observed in another study which showed that younger individuals were more likely to accept decriminalization of medical marijuana [19, 21]. This result, however, contradicts other findings which suggested that age was not a significant factor in acceptance towards decriminalization of medical marijuana [22].

The majority of the respondents who participated in this study were male (76.2%). The ratio of male and female respondents in this study did not correlate with the baseline gender ratio in Selangor’s population which shows only a slightly higher number of males (52%) compared to females. The difference could be explained by males having a higher tendency to respond to the survey compared to females. Moreover, the analysis of the data shows that gender is a significant factor associated with acceptance towards decriminalization of medical marijuana (p = 0.005). The findings are similar with other studies which found that males were significantly more likely to accept decriminalization of medical marijuana than females [17, 19, 21].

The distribution of the respondent ethnicities in this study was observed to be not proportionate with the baseline distribution of the Malay (55%), Chinese (24%), Indian (11%), and other (10%) ethnicities in Selangor. In this study, the majority of the respondents were Malay (82.5%), followed by Chinese (8.4%), Indian (4.3%), and others (4.8%). Nevertheless, the analysis from this study shows that ethnicity is a significant factor associated with decriminalization of medical marijuana (p = 0.012) which is similar with the findings by a study in the United States [21]. The overall acceptance towards decriminalization of medical marijuana in this study is relatively high considering the findings in the United States which showed that Asians were more likely to be unsure or rejected decriminalization of medical marijuana [22]. However, using multiple regression analysis, ethnicity was found to be not a significant predictor for acceptance towards decriminalization of medical marijuana.

All of the respondents who participated in this study had received formal education to at least primary school level. The results from this study show that acceptance towards decriminalization of medical marijuana was observed to be highest among respondents who held a first degree (71.4%), followed by those with Master’s/PhD qualifications (66.7%), STPM/Matriculation/Diploma holders (66.4%), and primary school leavers (33.3%). Chi-square test analysis of the data shows that level of education is significantly associated with acceptance towards decriminalization of medical marijuana (p = 0.011) as similarly reported by other studies that suggested those who had graduated from college had a significantly higher level of support towards a policy that decriminalized medical marijuana than those who had not [18, 21].

Almost half of the participants in the sample of this study are working with a private company (41.1%), followed by self-employed (29.4%), or a government servant (14.9%), unemployed (10%), and student (4.1%). The association analysis of the data shows that employment status is a significant factor in acceptance towards decriminalization of medical marijuana (p = 0.001). This finding contradicts the findings reported by other studies that showed employment status was not a significant factor associated with acceptance towards decriminalization of medical marijuana [23–25]. Further multiple logistic regression analysis shows that employment status is not a significant predictor for the dependent variable in this study.

The household monthly income for the respondents involved in the study has been categorized into B40 (44.0%), M40 (35.7%), and T20 (19.9%) based on the categorization of household monthly income for Malaysia as set by the Department of Statistics Malaysia (DOSM) in the Household Income and Basic Amenities Survey Report 2019 [26]. From the collected data, the distribution of the respondents based on household monthly income was observed to approximate Malaysia’s national standard for B40 (40%), M40 (40%), and T20 (20%). Analysis of this data shows that household monthly income is not significantly associated with acceptance towards decriminalization of medical marijuana.

History of smoking status ranging from ever smoked, current smoker, and those who have given up smoking has been shown to be a significant factor associated with acceptance towards decriminalization of medical marijuana policy [27]. From the obtained data, more than half of the respondents who participated in this study reported that they had smoked before (68.6%) and this status was observed to be significantly associated with acceptance towards decriminalization of medical marijuana from initial data analysis (p < 0.001).

The analysis of respondents who have given up smoking also suggests a significant association between the factor with acceptance towards decriminalization of medical marijuana. The finding is consistent with another study that suggested current cigarette smokers had more support for decriminalization of medical marijuana [22]. Subsequent analysis by logistic regression shows that factors of having ever smoked, current cigarette smoker, and those who have already given up smoking are not significant predictors of acceptance towards decriminalization of medical marijuana.

For alcohol drinking status, history of ever drank, current drinker, and given up alcohol were found to be not significant as factors associated with acceptance towards decriminalization of medical marijuana except for history of or current use of alcohol. The findings from this study are similar to other studies, which suggest that there is no significant relationship between alcohol drinking status of ever drank (p = 0.050), current alcohol drinker (p = 0.281), and given up alcohol (p = 0.145) with acceptance towards decriminalization [17, 22, 24]. Subsequent analysis by multiple logistic regression also shows that ever drank, current alcohol drinker, and given up alcohol are not significant predictors for acceptance towards decriminalization policy for medical marijuana.

In the analysis of the substance use status of respondents to this study, the number of respondents who reported themselves as have ever used drugs (48.1%) is relatively high considering the data by NADA (2019) which showed that Selangor had the lowest number of substance abusers and drug addicts per estimated total population compared with other states in Malaysia with a ratio of 0.21 or 210 substance abusers and drug addicts per 100,000 residents. However, the reported high number could be because the study did not differentiate the intention of use of the substances. The findings were initially observed on the reported data of respondents who answered “Yes” on the ever-used substance question, followed by giving information on the types of substance used, but declared themselves as “Never used substance” when asked “Have you given up illicit substance use?”. The explanation that might fit such responses is the respondents had received the substance from health care facilities as part of their emergency treatment or clinical management. The situation was also found to be similar if they answered “Yes” or “No” to the question.

Further analysis by multiple logistic regression shows that ever used substance and current substance use are significant predictors for acceptance towards decriminalization of medical marijuana.

Analysis of the online media use status shows that a majority of the respondents were online social media users (98.7%). This was unexpected given the study instrument was a self-administrated questionnaire that was distributed to the eligible respondents via WhatsApp or Telegram application which are considered to be social media platforms. A possible explanation for such responses is the questionnaire had been answered by a close acquaintance or family member on behalf of the respondents. Other possible explanations are the respondents might have a different interpretation of what online media is, for example, only Facebook, Instagram, and Twitter are considered as social media but not WhatsApp or Telegram or online portal news which may result in such negative response.

The results from the initial analysis of this study show that there is no significant relationship between online media users and non-users with acceptance towards decriminalization of medical marijuana (p = 0.092). However, a significant association was observed for exposure to medical marijuana-related content (p < 0.001) and hours of online media engagement (p < 0.001) with acceptance towards decriminalization. The significant result for this item is similar to the results of other studies in terms of a higher level of acceptance among those with higher mean of hours spent on online media [28, 29]. Further analysis by multiple logistic regression on online media use status shows that online media user, exposure to medical-marijuana related content, and hours of online media engagement are not significant predictors for acceptance towards decriminalization of medical marijuana.

Perception of medical marijuana that has been assessed in this study consists of the components of perceived risk of medical marijuana, perceived risk of medical marijuana use, and perceived risk of approval of medical marijuana use. The Chi-square test analysis of this study shows that there is a significant association between perceived risk of medical marijuana (p < 0.001), perceived risk of harm of medical marijuana use (p < 0.001), and perceived approval of use (p < 0.001). Further multiple regression analysis also shows that all the components of perception of marijuana are significant predictors associated with the acceptance towards decriminalization of medical marijuana among the adult population in Selangor, Malaysia.

The predictors for acceptance towards decriminalization of medical marijuana are perceived high approval of medical marijuana use (aOR = 7.023, p < 0.001, 95%CI = 3.534,13.955), perceived low risk of medical marijuana (aOR = 5.716, p < 0.001, 95%CI = 2.828,11.554), perceived low risk of harm of medical marijuana use (aOR = 3.480, p = 0.001, 95%CI = 1.702,7.114), current substance use (aOR = 2.264, p = 0.050, 95%CI = 1.001,5.118), and ever used substance (aOR = 2.005, p = 0.004, 95% CI = 0.054,0.576). The strongest predictor for reporting an acceptance towards decriminalization of medical marijuana policy is perceived high approval of medical marijuana use, recording an odds ratio of 7.023. This indicates that respondents who perceive high approval of medical marijuana use are 7 times more likely to report an acceptance towards decriminalization of medical marijuana than those who perceive low approval of use, controlling for all other factors in the model.

The adjusted odds ratio for ever used substance is 2.005, indicating that respondents who have ever used substance are 2 times more likely to report acceptance towards decriminalization of medical marijuana, controlling for all other factors in the model. The adjusted odds ratio for current substance use is 2.264, indicating that respondents who have a history of substance use in the last 30 days are more than 2 times more likely to report acceptance towards decriminalization of medical marijuana in comparison, controlling for all other factors in the model. The adjusted odds ratio of perceived risk of medical marijuana is 5.716, indicating that respondents who have low perceived risk of medical marijuana use are about 6 times more likely to report acceptance towards decriminalization of medical marijuana, controlling for all other factors in the model. The adjusted odds ratio of perceived risk of harm of medical marijuana use is 3.48, indicating that respondents who have a low perceived risk of harm of medical marijuana use are over 3 times more likely to report acceptance towards decriminalization of medical marijuana, controlling for all other factors in the model.

### Limitations

The study only looked into the association between sociodemographics, socioeconomics, lifestyle, and perception towards medical marijuana with the acceptance towards decriminalization of medical marijuana but did not assess the dynamic relationships between the evaluated variables due to the cross-sectional study design. This study only represents acceptance towards decriminalization of medical marijuana among the adult population in Selangor which is a highly urban state and may be associated with a high level of awareness towards medical marijuana policy. As the questionnaire was shared with the respondents through the WhatsApp and Telegram applications, there was a tendency for selection sampling bias as those who accept decriminalization of medical marijuana had a tendency to respond to the survey.

### Strength

This questionnaire was prepared in English and Malay to suit respondents’ language preferences and was validated. The respondents could answer the questionnaire privately and freely without the need to worry about any legal repercussions or a breach in sensitive information such as a history of illegal substance use. All data responses are protected, private, and confidential. This is the strength of the study that contributed to the respondents giving truthful responses in the questionnaire, which may explain the unexpectedly high prevalence among respondents who reported themselves as having previously used substances in the data sample.

## Conclusion

The results of the study show that the current acceptance level towards decriminalization of medical marijuana among the adult population in Selangor, Malaysia is considerably high. However, the acceptance is mainly among those who are exposed to the substance and those who perceive low risk of medical marijuana. Thus, further exploration on this phenomenon is needed, specifically by increasing the sample size and expanding the study location to other states in Malaysia.

### Recommendations

Based on the findings in this study that shows a high acceptance towards decriminalization of medical marijuana among the adult population in Selangor, Malaysia, it is recommended that the exploration of the acceptance level be extended further to the adult population at the national level and the acceptance level monitored periodically in preparation for dealing with the issue of medical marijuana in the country. A generally high acceptance rate towards decriminalization, with perceived low risk of medical marijuana, low risk of harm from medical marijuana use, and high approval of use of medical marijuana among the general population should be seen as an opportunity to be explored in terms of policy reconciliation, clinical, public health and agricultural research, expansion of medical services, social construct, as well as of crime and economic impact to the country.

As the issue of medical marijuana is a medical-related one, a focused study involving medical doctors, pharmacists, medical assistants, nurses, and other health care workers in a public or private setting may provide a different perspective from the point of view of the medical service provider regarding the acceptance of medical marijuana as a medical treatment. Moreover, decriminalization, alongside the right information and continuous education, is one of the approaches that could be considered in dealing with the medical marijuana issue in Malaysia.

## Supporting information

S1 File QUESTIONNAIRE medical marijuana. (DOCX)
