# Supplementary material for: Acceptance towards decriminalization of medical marijuana among adults in Selangor, Malaysia
Source: PLoS One. 2022 Feb 10;17(2):e0262819. doi: 10.1371/journal.pone.0262819 (PMC8830660; doi:10.1371/journal.pone.0262819)
Supplement: S1 File — (DOCX) [file pone.0262819.s001.docx]

| Nombor Kod*(Code no.)* |  |  |  |  |
| --- | --- | --- | --- | --- |

APPENDIX

#### BORANG KAJI SELIDIK

#### *Questionnaire*

**TAJUK KAJIAN:** PENERIMAAN DI KALANGAN PENDUDUK DEWASA DI SELANGOR PADA TAHUN 2021 TERHADAP DEKRIMINALISASI PENGGUNAAN GANJA UNTUK TUJUAN PERUBATAN DI MALAYSIA

***RESEARCH TITLE:*** *ACCEPTANCE TOWARDS DECRIMINALIZATION OF*

*MEDICAL MARIJUANA AMONG ADULT IN SELANGOR, MALAYSIA*

**PENYELIDIK (*RESEARCHER*)**

Dr Mohd Hafizuddin bin Mahfot (UPM)

**PENYELIA (*SUPERVISOR*)**

Dr Rahmat bin Dapari (UPM)

**PENYELIA BERSAMA (CO-SUPERVISOR)**

Dr Ahmad Iqmer Nashriq bin Mohd Nazan (UPM)

#### Borang ini mengadungi bahagian seperti berikut:

*This form has sections divided as below:*

| 1 | Faktor-faktor sosiodemografik, sosioekonomik dan gaya hidup.*Sociodemographic, socioeconomic and lifestyle factors* |
| --- | --- |
| 2 | Persepsi risiko terhadap ganja perubatan *Perceived risk of medical marijuana* |
| 3 | Penerimaan terhadap dekriminalisasi ganja untuk tujuan perubatan di Malaysia *Acceptance towards decriminalization of medical marijuana in Malaysia* |

Maklumat dalam borang kaji selidik ini hanyalah untuk kegunaan penyelidikan semata-mata dan identiti adalah sulit. *(The information in this questionnaire is solely for research purpose and your identity remain strictly confidential).*

Tarikh (*date*): ____________

Arahan (*Intruction)*

Sila jawab semua soalan disetiap muka surat borang kaji selidik. *(Please answer all the question in each page of the questionnaire)*

**BAHAGIAN 1: FAKTOR-FAKTOR SOSIODEMOGRAFIK, SOCIOEKONOMIK DAN GAYA HIDUP *(SECTION 1: SOCIODEMOGRAPHIC, SOCIOECONOMIC AND LIFESTYLE FACTORS)***

| Bahagian di bawah mengandungi faktor-faktor berkaitan sosiodemografik, sosioekonomik, dan gaya hidup. Sila tandakan (✔) pada SATU pilihan jawapan yang paling bersesuaian dengan anda melainkan pada penyataan yang bertanda (*). Bagi soalan penyataan, anda diminta untuk menuliskan jawapan diruang yang disediakan.  *The following section contain sociodemographic, socioeconomic and lifestyle factors. Please tick (✔) on an answer that suits you the most except for statement with (*) mark. For statement question, your may write your answer in the column provided.* | | | | | | | | | | | | | | | | | |
| --- | --- | --- | --- | --- | --- | --- | --- | --- | --- | --- | --- | --- | --- | --- | --- | --- | --- |
| **FAKTOR-FAKTOR SOSIODEMOGRAFIK *(SOCIODEMOGRAPHIC FACTORS)*** | | | | | | | | | | | | | | | | | |
| **Umur:**  ***(Age)*** | |  | | **Jantina:**  ***(Gender)*** | | | | | | | |  | Lelaki  *(Male)* | | | | |
|  |  |  |  |  |  |  |  |  |  |  |  |  | Perempuan  *(Female)* | | | | |
| **Bangsa:**  ***(Race)*** | |  | Melayu  *(Malay)* | **Pendidikan formal tertinggi:**  ***(Highest formal education)*** | | | | | | | |  | Sekolah Rendah  *(Primary school)* | | | | |
|  |  |  | Cina  *(Chinese)* |  |  |  |  |  |  |  |  |  | Sekolah Menengah  *(Secondary school)* | | | | |
|  |  |  | India  *(Indian)* |  | | Ijazah/ Sarjana Muda  *(First degree)* | | | | | |  | SPM/STPM/ Matrikulasi/Diploma  *(SPM/STPM/matriculation/ Diploma)* | | | | |
|  |  |  | Lain-lain:……….  *(Others)* |  | | Master/Sarjana/PhD  *(Second and third degree)* | | | | | |  | Tidak bersekolah  *(No formal education)* | | | | |
| **FAKTOR-FAKTOR SOSIOEKONOMIK *(SOCIOECONOMIC FACTORS)*** | | | | | | | | | | | | | | | | | |
| **Status pekerjaan:**  ***(Employment status)*** | | | | | | | | |  | Pelajar  *(Student)* | | | | | | | |
|  | Bekerja kerajaan  *(Government servant)* | | | | | | | |  | Tidak bekerja  *(Unemployed)* | | | | | | | |
|  | Bekerja swasta  *(Working with private company)* | | | | | | | |  | Bekerja sendiri  *(Self-employed)* | | | | | | | |
| **Pendapatan bulanan persendirian:**  ***(Monthly individual income)*** | | | | |  | | | | **Pendapatan bulanan seisi rumah:**  ***(Monthly household income)*** | | | | | |  | | |
| **FAKTOR-FAKTOR GAYA HIDUP *(LIFESTYLE FACTORS)*** | | | | | | | | | | | | | | | | | |
| **Adakah anda pernah merokok?**  ***(Have you ever smoked)*** | | | | | | |  | Ya  *(Yes)* | | | **Adakah anda ada merokok dalam tempoh 30 hari yang lalu?**  ***(Have you smoked in the past 30 days?)*** | | | | |  | Ya  *(Yes)* |
|  |  |  |  |  |  |  |  | Tidak  (No) | | |  |  |  |  |  |  | Tidak  *(No)* |
| **Adakah anda telah berhenti merokok?**  ***(Have you given up smoking?)*** | | | | | | |  | Ya  (Yes)  Tidak  (No) | | | **Jika ya, nyatakan tarikh berhenti merokok:**  ***(If yes, state date given up smoking)*** | | |  | | | |
|  |  |  |  |  |  |  |  |  |  |  |  |  |  |  |  |  |  |
| **Adakah anda pernah meminum arak?**  ***(Do you ever drink alcohol?)*** | | | | | | |  | Ya  *(Yes)* | | | **Adakah anda ada meminum arak dalam tempoh 30 hari yang lalu?**  ***(Have you drunk alcohol in the past 30 days?)*** | | | | |  | Ya  *(Yes)* |
|  |  |  |  |  |  |  |  | Tidak  *(No)* | | |  |  |  |  |  |  | Tidak  *(No)* |
| **Adakah anda telah berhenti meminum arak?**  ***(Have you given up alcohol drinking?)*** | | | | | | |  | Ya  (Yes) | | | **Jika ya, nyatakan tarikh berhenti minum arak:**  ***(If yes, state date given up alcohol)*** | | |  | | | |
|  |  |  |  |  |  |  |  | Tidak  (No) | | |  |  |  |  |  |  |  |

| **Adakah anda pernah menggunakan dadah terlarang seperti opiate, methamphetamine, ganja, amphetamine, ketum atau lain-lain jenis dadah?**  ***(Do you ever use illicit substance e.g opiod, methamphetamine, marijuana, amphetamine, kratom or others?)*** | | | | | |  | Ya  (Yes) |
| --- | --- | --- | --- | --- | --- | --- | --- |
|  |  |  |  |  |  |  | Tidak  (No) |
| ***Apakah jenis dadah terlarang yang pernah anda gunakan?**  ***(What type of illicit drugs do you ever use?** | |  | Methamphetamine - kristal  (Methamphetamine - crystalline) | | | | |
|  | Amphetamine  (Amphetamine) |  | Methamphetamine - tablet  (Methamphetamine - tablet) | | | | |
|  | Ganja  (Marijuana) |  | Ketum  (Kratom) | | | | |
|  | Opiat  (Opioid) |  | Lain-lain, nyatakan:  (Others, please state) |  | | | |
| **Adakah anda ada menggunakan dadah terlarang dalam tempoh 30 hari yang lalu?**  **(Have you used illicit substance in the past 30 days?)** | | | | | |  | Ya  (Yes) |
|  |  |  |  |  |  |  | Tidak  (No) |
| **Adakah anda pengguna media dalam talian?**  **(Are you an online media user?)** | | | | | |  | Ya  (Yes) |
|  |  |  |  |  |  |  | Tidak  (No) |
| **Adakah anda pernah terdedah kepada kandungan ganja perubatan melalui media dalam talian?**  **(Have you ever exposed to medical marijuana-related content through online media?)** | | | | | |  | Ya  (Yes) |
|  |  |  |  |  |  |  | Tidak  (No) |
| **Berapa jam sehari anda gunakan untuk melayari media dalam talian? Nyatakan bilangan jam:**  **(How many hours do you spend on online media per day? State number of hours)** | | | | |  | | |

**BAHAGIAN 2: PERSEPSI RISIKO TERHADAP GANJA PERUBATAN *(SECTION 2: PERCEIVED RISK OF MEDICAL MARIJUANA)***

| Bahagian di bawah mengandungi kenyataan yang mana sesetengah orang akan bersetuju dan sesetengah orang tidak akan bersetuju. Sila tandakan (✔) sejauh mana anda bersetuju dengan kenyataan tersebut berdasarkan kepada skala berikut:  *The section below contain a number of statements with which some people agree and others disagree. Please tick (✔) on how much you agree with the statement based on the following scale:* | | | | | | | | | |
| --- | --- | --- | --- | --- | --- | --- | --- | --- | --- |
| Bil/ (No) | Kenyataan  (Statement) | | 0 | 1 | | 2 | | 3 | 4 |
|  |  |  | Sangat tidak setuju  *(Strongly disagree)* | Tidak setuju  *(Disagree)* | | Tidak pasti  *(Unsure)* | | Setuju  *(Agree)* | Sangat setuju  *(Strongly agree)* |
| 1. | Penggunaan ganja perubatan adalah berbahaya  *Medical marijuana use is harmful.* | |  |  | |  | |  |  |
| 2. | Ganja perubatan adalah bahan yang menyebabkan ketagihan.  *Medical marijuana is addictive.* | |  |  | |  | |  |  |
| 3. | Penggunaan ganja perubatan menyebabkan risiko kesihatan mental yang serius  *Using medical marijuana poses serious mental health risk* | |  |  | |  | |  |  |
| 4. | Penggunaan ganja perubatan menyebabkan risiko kesihatan fizikal yang serius  *Using medical marijuana poses serious physical health risk* | |  |  | |  | |  |  |
| Bahagian yang berikutnya mengandungi kenyataan yang mana sesetengah orang memberi penilaian risiko yang berbeza. Sila tandakan (✔) pada penilaian risiko yang sesuai dengan kenyataan yang diberikan berdasarkan kepada skala berikut:  *The following section contain a number of statements in with which the risk perceived differently by different people. Please tick (✔) on the risk you think appropriate for the statement based on the following scale:* | | | | | | | | | |
| Bil/ (No) | Kenyataan  (Statement) | | 0 | 1 | 2 | | 3 | | 4 |
|  |  |  | Tiada risiko  *(No risk)* | Risiko rendah  *(Slight risk)* | Tidak pasti  *(Unsure)* | | Risiko sederhana  *(Moderate risk)* | | Risiko tinggi  *(High risk)* |
| 1. | Apakah risiko seseorang itu memudaratkan diri sendiri secara fizikal atau apa-apa cara lain sekiranya mereka…   1. *Mencuba ganja perubatan sekali atau dua kali*   *How much do you think people risk harming themselves (physically or in other ways), if they...*   1. *Try medical marijuana once or twice* | |  |  |  | |  | |  |
| 2. | Apakah risiko seseorang itu memudaratkan diri sendiri secara fizikal atau apa-apa cara lain sekiranya mereka…   1. *Menggunakan ganja perubatan sekali-sekala*   *How much do you think people risk harming themselves (physically or in other ways), if they...*   1. *Use medical marijuana occasionally* | |  |  |  | |  | |  |
| 3. | Apakah risiko seseorang itu memudaratkan diri sendiri secara fizikal atau apa-apa cara lain sekiranya mereka…   1. *Mengguna ganja perubatan secara kerap*   *How much do you think people risk harming themselves (physically or in other ways), if they...*   1. *Use medical marijuana regularly* | |  |  |  | |  | |  |
| Bahagian yang berikutnya mengandungi kenyataan yang mana sesetengah orang memberi penilaian penerimaan yang berbeza. Sila tandakan (✔) pada penilaian penerimaan yang sesuai dengan kenyataan yang diberikan berdasarkan kepada skala berikut:  *The following section contain a number of statements in with which the risk perceived differently by different people. Please tick (✔) on the risk you think appropriate for the statement based on the following scale:* | | | | | | | | | |
| Bil/ (No) | | Kenyataan  (Statement) | 0 | 1 | 2 | | 3 | | 4 |
|  |  |  | Sangat terima  *(Strong yes)* | Terima  *(Yes)* | Tidak pasti  *(Unsure)* | | Tidak terima  *(No)* | | Sangat tidak terima  *(Strong no)* |
| 1. | | Adakah anda menerima seseorang (berumur 18 tahun dan ke atas) melakukan perkara berikut…   1. *Mencuba ganja perubatan sekali atau dua kali*   *Do you disapprove of people (who are 18 or older) doing each of the following?...*   1. *Try medical marijuana once or twice* |  |  |  | |  | |  |
| 2. | | Adakah anda menerima seseorang (berumur 18 tahun dan ke atas) melakukan perkara berikut…   1. *Menggunakan ganja perubatan sekali-sekala*   *Do you disapprove of people (who are 18 or older) doing each of the following?...*   1. *Use medical marijuana occasionally* |  |  |  | |  | |  |
| 3. | | Adakah anda menerima seseorang (berumur 18 tahun dan ke atas) melakukan perkara berikut…   1. *Menggunakan ganja perubatan secara kerap*   *Do you disapprove of people (who are 18 or older) doing each of the following?...*   1. *Use medical marijuana regularly* |  |  |  | |  | |  |

**BAHAGIAN 3: PENERIMAAN TERHADAP DEKRIMINALISASI GANJA PERUBATAN IN MALAYSIA *(SECTION 3: ACCEPTANCE TOWARDS DECRIMINALIZATION OF MEDICAL MARIJUANA IN MALAYSIA)***

| Bahagian yang berikutnya mengandungi kenyataan yang mana sesetengah orang akan bersetuju dan sesetengah orang tidak akan bersetuju. Sila tandakan (✔) sejauh mana anda bersetuju dengan kenyataan tersebut berdasarkan kepada skala berikut:  *The following section contain a number of statements with which some people agree and others disagree. Please tick (✔) on how much you agree with the statement based on the following scale:* | | | | | |
| --- | --- | --- | --- | --- | --- |
| Kenyataan  (Statement) | 0 | 1 | 2 | 3 | 4 |
|  | Sangat tidak setuju  *(Strongly disagree)* | Tidak setuju  *(Disagree)* | Tidak pasti  *(Unsure)* | Setuju  *(Agree)* | Sangat setuju  *(Strongly agree)* |
| Saya menerima rawatan ganja perubatan yang berasaskan kepada bukti kajian klinikal.  *I accept the use of medical marijuana that is evidence based from clinical research.* |  |  |  |  |  |
| Saya menerima rawatan ganja perubatan yang berasaskan kepada bukti kajian klinikal digunakan oleh keluarga atau kenalan rapat.  *I accept the use of medical marijuana that is evidence based from clinical research among my family members or close acquaintance.* |  |  |  |  |  |
| Saya setuju penggunaan ganja untuk tujuan perubatan memerlukan khidmat nasihat, preskripsi, dan pemantauan daripada doktor perubatan terlatih  *I agree marijuana use for medical purposes requires consultation, prescription and monitoring from trained medical doctor* |  |  |  |  |  |
| Saya setuju penggunaan ganja untuk tujuan perubatan adalah selamat kepada kesihatan mental dan fizikal  *I agree medical marijuana is safe for mental and physical health* |  |  |  |  |  |
| Saya setuju dengan dekriminalisasi ganja untuk tujuan perubatan di Malaysia.  I agree on decriminalization of medical marijuana in Malaysia. |  |  |  |  |  |
